# Nonanatomic resection is not inferior to anatomic resection for primary intrahepatic cholangiocarcinoma: A propensity score analysis

**DOI:** 10.1038/s41598-018-35911-5

**Published:** 2018-12-12

**Authors:** B. Li, J. L. Song, Y. Aierken, Y. Chen, J. L. Zheng, J. Y. Yang

**Affiliations:** Department of Liver Surgery & Liver Transplantation Center, West China Hospital of Sichuan University, Chengdu, 610041 Sichuan Province China

## Abstract

Whether anatomic resection (AR) achieves better outcomes than nonanatomic resection (NAR) in patients with primary intrahepatic cholangiocarcinoma (ICC) is unclear. Data were retrieved for all consecutive patients who underwent liver resection for primary ICC from January 2007 to July 2017. The prognoses of the patients without direct invasion to contiguous organs or extrahepatic metastasis who underwent AR or NAR were compared. 85 patients underwent AR, and 65 patients underwent NAR. operation time were slightly decreased in the NAR group. The risk of Clavien-Dindo classification (CDC) IV in the AR group was significant higher than that in the NAR group. Cox regression analysis showed lymph node metastasis and adjuvant therapy were significant prognostic factors for overall survival (OS) and disease-free survival (DFS), respectively. After 1:1 propensity score matching (PSM), 29 pairs of patients were compared. The survival curves showed the NAR group had slightly improved DFS and OS than the AR group before and after matching. Thus, we conclude NAR was not inferior to AR in improving the survival outcomes for patients with primary solitary ICC lesions without direct invasion to contiguous organs or extrahepatic metastasis. Furthermore, patients may benefit from NAR.

## Introduction

Intrahepatic cholangiocarcinoma (ICC) is the second most common malignant hepatic tumour and accounts for 5% to 30% of all primary liver malignancies^1–4^. Hepatic resection remains the first-line therapeutic option for cure of primary malignant liver tumors, and it is a surgical option for a broad range of patients with various stages of disease, as long as the patient has been with adequate liver remnant. The extent of resection for primary malignant liver tumors has been a topic of much interest. Anatomic resection (AR) is defined as the removal of a “hepatic segment or sub-segment, which include tumorbearing portal tributaries as well as major branch of the portal vein and hepatic artery”^5^. In theory, AR may be more effective in removing the entire tumor burden including possible satellites, nodules, as well as any high risk area of micro-portal invasion and intra-hepatic metastasis. On the other hand, parenchymal sparing or non-anatomic resection (NAR) offers less extensive liver resection that, in turn, may be associated with lower perioperative morbidity, as well as a lower incidence of an inadequate future liver remnant (FLR) and liver insufficiency^6^. Intrahepatic recurrence is regarded as occurring primarily through vascular invasion for patients with hepatocellular carcinoma (HCC), with AR considered theoretically effective for eradicating these intrahepatic metastases^5,7^. Thus, Makuuchi *et al*.^5^ originally introduced comparative studies AR vs NAR for single HCC lesions, and the potential benefit of AR for HCC lesions has been indicated^8–12^. For example, a systematic review and meta-analysis^13^ reported AR was associated with a disease-free survival (DFS) benefit at 1-, 3- and 5- years (p = 0.002, p = 0.004 and p < 0.0001, respectively) and also was associated with a decreased risk of death at 5-years (p = 0.01); however, an original study^14^ showed there was no difference in overall recurrence-free survival between the AR and NAR groups (P = 0.290). To our knowledge, the 5-year OS rate of ICC patients is reported from 30% to 35% after hepatectomy^7^. However, few studies have investigated the clinical outcomes between AR and NAR for ICC. In this study, we aimed to compare the perioperative outcomes and prognoses of patients between the AR and NAR groups with primary solitary ICC lesions without direct invasion to contiguous organs or extrahepatic metastasis using a one to one propensity score matching (PSM) analysis.

## Results

### Baseline patient and Clinicopathological characteristics

Patients were followed until the date of death or the final date of the study, December 30, 2017. A total of 150 patients underwent hepatectomy for primary solitary ICC lesions without direct invasion to contiguous organs or extrahepatic metastasis. These patients were followed for 3 to 107 months (median 12 months). The majority of patients were male (n = 93, 62.0%). More than half of the patients presented with a tumour size ≤5 cm (n = 80, 53.3%). Of note, half of the patients were positive for hepatitis B virus surface antigen (HBsAg) (n = 76, 50.7%), and presented with cirrhosis (n = 83, 55.3%). Only 9.3% patients had intrahepatic biliary stones (n = 14). Specifically, vascular invasion, periductal invasion, and perineural invasion were only observed in 17.3% (n = 26), 2.7% (n = 4), and 4.7% (n = 7) of tumours, respectively. Poorly differentiated lesions were found in 34.7% (n = 52) of patients, metastatic nodal disease was observed in 12.7% (n = 19) of these patients, and a positive resection margin was found in 8 (5.3%) patients. Regarding liver function, as defined by the Child-Pugh classification, 131 (87.3%) patients were class A, and 7 (4.7%) were class B; Of these 150 patients, 85 (56.7%) underwent AR, and 65 (43.3%) underwent NAR. Patients undergoing NAR were younger, more likely to be positive for HBsAg (NAR vs. AR; 60.0% VS 43.5%, P = 0.046), and more often presented with cirrhosis (NAR vs. AR; 66.2% VS 47.1%, P = 0.02). NAR was more frequently performed among patients who had small tumors. Patients undergoing NAR were also more likely to have advanced disease characterized by poor tumor differentiation (Table 1). Interestingly, patients who underwent a NAR were more likely to have a microscopically positive margin than patients who underwent AR (p = 0.001). Post-operatively, of these patients, 31 (40.8%) patients received antiviral therapy, and 43 (28.7%) patients received adjuvant therapy, including adjuvant chemotherapy, radiofrequency ablation, transcatheter arterial chemoembolization, and re-operation (Table 1). Notebly, NAR had more tumour distributing in the right liver than AR (NAR vs. AR; 73.8% VS 36.5%, P < 0.001) (Supplementary Table S1).

**Table 1:** Demographic and clinicopathological characteristics of ICC.

	All patients			Propensity score-matched patients		
	AR group	NAR group	P-value	AR group	NAR group	P-value
	n = 85	n = 65		n = 29	n = 29	
**Background characteristics**						
Sex, male, n (%)	49 (57.6)	44 (67.7)	0.209	16(55.2)	16(55.2)	1
Age, median (range, yr)	59 (27–87)	54 (27–76)	0.043*	61 (27–87)	57 (36–72)	0.533
HBsAg, n (%)	37 (43.5)	39 (60.0)	0.046*	16 (55.2)	14 (48.3)	0.599
intrahepatic biliary stones,n(%)	11 (12.9)	3 (4.6)	0.082	1 (3.4)	2 (6.9)	1
Child-Pugh class B, n (%)	4 (4.7)	3 (4.6)	0.236	4 (13.8)	2 (6.9)	0.225
Cirrhosis, n (%)	40 (47.1)	43 (66.2)	0.02*	16 (55.2)	17 (58.6)	0.791
**Tumour-related factors**						
CEA, ng/mL, median (range)	3 (0–571)	2 (0–322)	0.257	1.85 (0–11.67)	2.54 (0–25.32)	0.239
CA19-9, U/mL, median (range)	28 (0–5533)	26 (0–844)	0.884	27.3 (0–449.1)	29.3 (0–497.7)	0.898
Tumour diameter, cm, median (range)	6 (2–13)	4.5 (1–13)	0.0003*	5 (2–13)	4.5 (1–12)	0.754
**Surgical factors**						
Operation time, min, median (range)	230(80–415)	190(89–477)	0.532	300 (110–415)	190 (89–477)	0.380
Blood loss, mL, median (range)	300(50–1500)	300(50–1300)	0.483	300 (100–1000)	200 (50–1300)	0.890
Intraoperative blood transfusion, n (%)	11(12.9)	10(15.4)	0.669	3 (10.3)	5 (17.2)	0.706
Postoperative blood transfusion, n (%)	13(15.3)	7(10.8)	0.419	4 (13.8)	3 (10.3)	1
Postoperative hospital stay,d,median(range)	8(1–39)	7(4–36)	0.733	7 (3–12)	7 (4–36)	0.097
antiviral therapy, n (%)	13 (15.3)	18(27.7)	0.063	4 (13.8)	6 (20.7)	0.487
Adjuvant therapy, n (%)	22 (25.9)	21(32.3)	0.388	7 (24.1)	8 (27.6)	0.764
**Pathological factors**						
Negative surgical margin, n (%)	85(100)	57(87.7)	0.001*	29 (100)	24 (82.8)	0.052
Tumour differentiation, poor, n (%)	23(27.1)	29(44.6)	0.025*	11 (37.9)	9 (31.0)	0.581
Vascular invasion, n (%)	17 (20)	9(13.8)	0.324	4 (13.8)	3 (10.3)	1
Perineural invasion, n (%)	6 (7.1)	1(1.5)	0.140	1 (3.4)	0	1
Periductal invasion, n (%)	2 (2.4)	2(3.1)	1	0	2 (6.9)	0.491
Lymph node metastasis, n (%)	11(12.9)	8(12.3)	0.908	4 (13.8)	1 (3.4)	0.352

* Indicates statistically significant.

AR, anatomic resection; CA19-9, carbohydrate antigen 19-9; CEA, carcinoembryonic antigen; HBsAg, hepatitis B virus surface antigen; ICC, intrahepatic cholangiocarcinoma; NAR, nonanatomic resection.

### Association of surgical procedure with Short and Long-Term Outcomes

There were no difference of Operation time, intraoperative blood loss, intra- and postoperative transfusion, and the length of postoperative hospital stay between the two groups (Table 1). Postoperative complications were stratified according to the Clavien-Dindo classification (CDC)^15^. Consequently, almost three of five patients (n = 40, 61.5%) developed a postoperative complications following NAR versus about three of four patients (n = 62, 72.9%) after AR (p = 0.138). Moreover, CDC IV was more common after AR versus NAR (p = 0.019), while CDC I, CDC II and CDC III were comparable (p = 0.138, p = 0.057 and p = 0.133, respectively, Table 2). Both groups were comparable with surgical technique-related (ascites, bile leakage, liver failure, cholangitis, and intra-abdominal bleeding), medical (pneumonia, pleural effusion, respiratory failure, and cardiac events), and infectious (systemic) complications (Table 2). No patients died in the hospital.

**Table 2:** Short- and long-term outcome of patients undergoing AR and NAR for intrahepatic cholangiocarcinoma.

	AR (n = 85)	NAR (n = 65)	*P* value
Postoperative complications	62 (72.9%)	40 (61.5%)	0.138
**Clavien-Dindo classification**			
I	62 (72.9%)	40 (61.5%)	0.138
II	21 (24.7%)	8 (12.3%)	0.057
III	4 (4.7%)	0	0.133
IV	7 (8.2%)	0	0.019*
V	0	0	1
Ascites	9 (10.6%)	12 (18.5%)	0.168
Bile leakage	0	2 (3.1%)	0.186
Cholangitis	9 (10.6%)	7 (10.8%)	0.972
Liver failure	2 (2.4%)	0	0.506
Intra-abdominal bleeding	2 (2.4%)	0	0.506
Pneumonia	9 (10.6%)	4 (6.2%)	0.339
Pleural effusion	3 (3.5%)	0	0.258
Respiratory failure	2 (2.4%)	0	0.506
Cardiac events	1 (1.2%)	1 (1.5%)	0.669
Systemic sepsis	0	2 (3.1%)	0.186
**Tumor recurrence**			
Intrahepatic	36 (42.4%)	22 (33.8%)	0.289
Extrahepatic	7 (8.2%)	5 (7.7%)	0.903
Intra- and extrahepatic	5 (5.9%)	6 (9.2%)	0.438

* indicates statistically significant.

**Figure 1:**
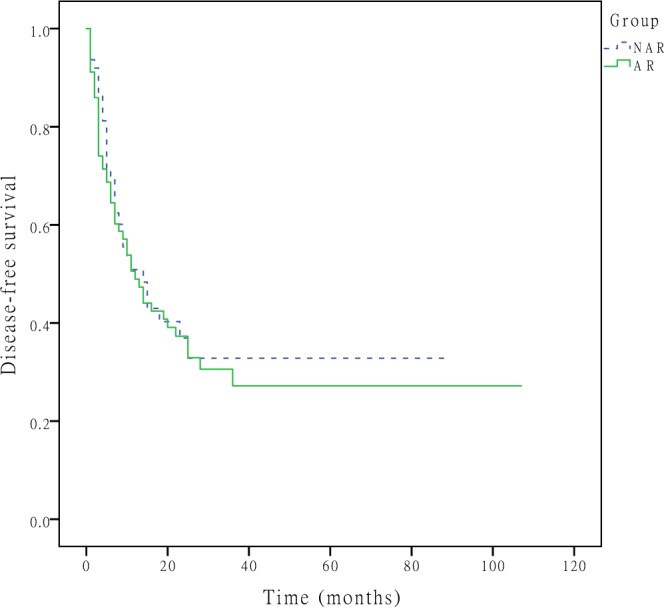
DFS curves after hepatectomy for primary solitary ICC patients who underwent NAR and AR. The 1-, 3-, and 5-year DFS rates were 50.9%, 32.8%, and 32.8% in the NAR group and 48.9%, 27.2%, and 27.2% in the AR group (P = 0.607), respectively.

**Figure 2:**
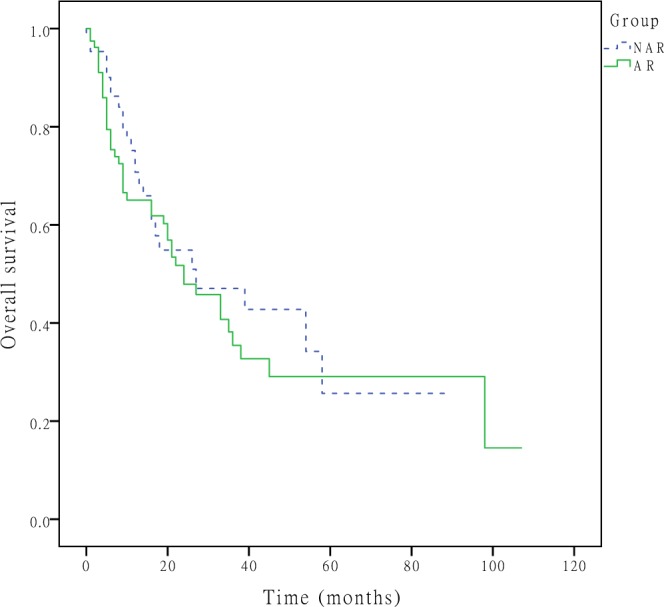
OS curves after hepatectomy for primary solitary ICC patients who underwent NAR and AR. The 1-, 3-, and 5-year OS rates were 75.2%, 47.0%, and 25.7% in the NAR group and 65.0%, 35.4%, and 29.1% in the AR group (P = 0.477), respectively.

**Figure 3:**
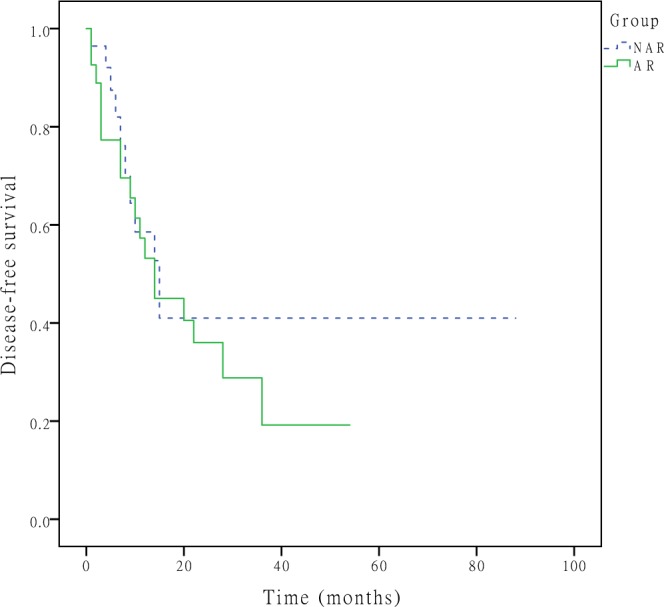
DFS curves after hepatectomy for primary solitary ICC patients who underwent NAR and AR after propensity score adjustment. the 1-, 3-, and 5-year DFS rates were 58.6%, 41.0% and 41.0% in the NAR group and 53.2%, 19.2% and 19.2% in the AR group (p = 0.370), respectively.

**Figure 4:**
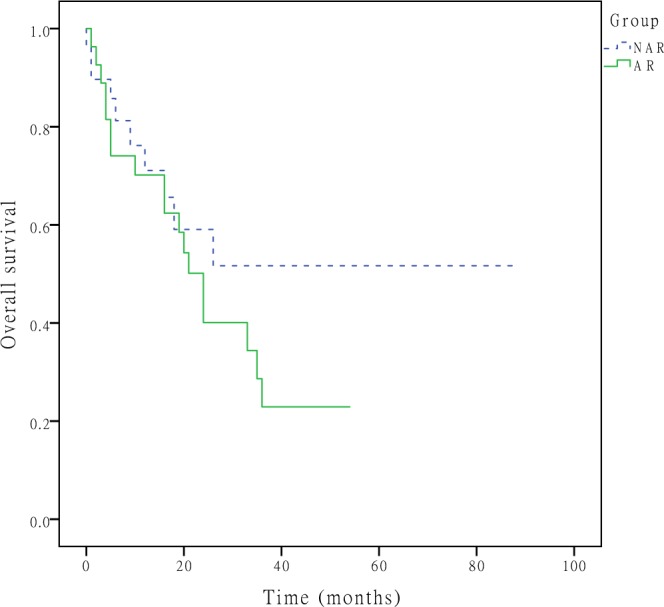
OS curves after hepatectomy for primary solitary ICC patients who underwent AR and NAR after propensity score adjustment. The 1-, 3-, and 5-year OS rates were 71.1%, 51.7% and 51.7% in the NAR group and 70.2%, 22.9% and 22.9% in the AR group (p = 0.229), respectively.

During the follow-up period, tumour recurrence developed in 79 (52.7%) patients, with recurrent lesions most commonly developing in the remnant liver (n = 58, 73.4%), and 70 patients (46.7%) died. The 1-, 3-, and 5-year DFS rates were 50.9%, 32.8%, and 32.8% in the NAR group and 48.9%, 27.2%, and 27.2% in the AR group (P = 0.607, Fig. 1), respectively. The 1-, 3-, and 5-year OS rates were 75.2%, 47.0%, and 25.7% in the NAR group and 65.0%, 35.4%, and 29.1% in the AR group (P = 0.477, Fig. 2), respectively. A Cox regression model was performed to identify risk factors associated with DFS and OS of these 150 patients. On multivariable analysis, adjuvant therapy was associated with better DFS (HR 0.757, [95% CI, 0.605–0.949], p = 0.016) (Table 3), lymph node metastasis was associated with worse OS (HR 1.972, [95% CI, 1.039–3.743], p = 0.038) (Table 4). To better account for any residual confounding due to case mix, a sensitivity analysis investigating the association of surgical procedure with DFS and OS was performed within a matched patient cohort. Patients were matched based on propensity scores obtained from a logistic regression model accounting for patient, operation and tumour characteristics. Based on this 1:1 propensity score matching (PSM) approach, 29 NAR patients and 29 AR patients were matched (Table 1). In the propensity model, both groups had equivalent DFS (the 1-, 3- and 5-year DFS rates (NAR vs. AR; 58.6%, 41.0% and 41.0% vs. 53.2%, 19.2% and 19.2%, p = 0.370, Fig. 3)) and OS (the 1-, 3- and 5-year OS rates (NAR vs. AR; 71.1%, 51.7% and 51.7% vs. 70.2%, 22.9% and 22.9%, p = 0.229, Fig. 4)).

**Table 3:** Clinicopathological factors associated with DFS in ICC patients.

Variable	Categorization	Univariate analysis	Multivariate analysis		
		*P* value	HR	95% CI	P value
Sex	female, male	0.929			
Age	≤56, >56	0.424			
HBsAg	negative, positive	0.035*			
intrahepatic biliary stones	negative, positive	0.421			
Tumour diameter, cm	≤5, >5	0.193			
Surgical procedure	nonanatomical, anatomical	0.607			
Complications		0.250			
No complication					
Clavien-Dindo grade I-II					
Clavien-Dindo grade III-IV					
Tumour differentiation	poor, others	0.116			
Vascular invasion	absent, present	0.074			
Perineural invasion	absent, present	0.896			
Periductal invasion	absent, present	0.852			
Surgical margin	negative, positive	0.658			
Lymph node metastasis	absent, present	0.119			
Pathological cirrhosis	absent, present	0.629			
antiviral therapy	no, yes	0.436			
Adjuvant therapy	no, yes	0.006*	0.757	0.605–0.949	0.016*

* indicates statistically significant.

DFS, disease-free survival; HBsAg, hepatitis B virus surface antigen; HR, hazard ratio; ICC, intrahepatic cholangiocarcinoma.

**Table 4:** Clinicopathological factors associated with OS in ICC patients.

Variable	Categorization	Univariate analysis	Multivariate analysis		
		*P* value	HR	95% CI	*P* value
Sex	female, male	0.526			
Age	≤56, >56	0.630			
HBsAg	negative, positive	0.026*			
intrahepatic biliary stones	negative, positive	0.501			
Tumour diameter, cm	≤5, >5	0.106			
Surgical procedure	nonanatomical, anatomical	0.477			
Complications		0.154			
No complication					
Clavien-Dindo grade I-II					
Clavien-Dindo grade III-IV					
Tumour differentiation	poor, others	0.266			
Vascular invasion	absent, present	0.007*			
Perineural invasion	absent, present	0.468			
Periductal invasion	absent, present	0.142			
Surgical margin	negative, positive	0.844			
Lymph node metastasis	absent, present	0.011*	1.972	1.039–3.743	0.038*
Pathological cirrhosis	absent, present	0.389			
antiviral therapy	no, yes	0.979			
Adjuvant therapy	no, yes	0.550			

* indicates statistically significant.

HBsAg, hepatitis B virus surface antigen; HR, hazard ratio; ICC, intrahepatic cholangiocarcinoma; OS, overall survival.

## Discussion

To date, partial hepatectomy remains the mainstay of curative treatment for ICC^16,17^. Unfortunately, prognosis after partial hepatectomy is unsatisfactory, with a high incidence of locoregional recurrence and/or extrahepatic metastasis and a low 5-year OS rate^18–20^. In the 1980 s, Muccuchi *et al*.^5^ proposed AR in hepatectomy for HCC. Previous studies^8–12^ showed the benefit of AR for single HCC lesions. Both ICC and HCC arise in the hepatic parenchyma. However, the extent of hepatic resection and its impact on outcomes among patients with ICC has not been well examined. Thus, whether the application of AR or NAR can improve the prognosis of ICC patients is worthy to investigate. Selection biases are inevitable in retrospective comparisons of postoperative outcomes in patients who underwent AR and NAR for ICC. To better account for any residual confounding due to case mix, we adjusted the clinicopathological characteristics of patients to eliminate as much selection bias as possible by excluding patients with multiple lesions, adjacent organ invasion, and extrahepatic metastasis. Furthermore, the clinicopathological characteristics of patients were adjusted by 1:1 PSM.

In the current study, patients who underwent AR tended to have larger tumors, the NAR group had more patients with positivity of HBsAg and liver cirrhosis. Previous study^6^ had pointed that the ideal surgical approach to hepatic resection of primary liver carcinoma should optimize locoregional control, yet preserve as much nontumorous hepatic parenchyma in the FLR. Theoretically, wider margins may offer a better chance of disease control, whereas a more limited resection allows for the preservation of hepatic parenchyma and mitigation of the risk for liver insufficiency^12,21,22^. Thus, patients with large tumors may, however, simply be more likely to undergo AR versus NAR as larger tumors may necessitate major resections to achieve an negative margin.

As mentioned above about the definition of AR and NAR. We can see the extent of liver resection is greater in AR than in NAR. Theoretically, the risk of postoperative liver failure is higher in AR, especially in patients who have comorbidities such as liver cirrhosis, the advantage of NAR is related to the postoperative liver function reserve. Decreases in albumin, prothrombin time, and cholinesterase during the first year after surgery were reported to be lower in patients who underwent NAR than in patients who underwent AR^23^. Thus, patients with hepatic tumours have concomitant injury to the nonmalignant liver parenchyma, a smaller range of resections can result in better restoration and/or preservation of the liver function^24–26^. Meanwhile, for patients with high risk of liver failure due to the low FLR, portal vein embolization can be performed to induce compensatory hypertrophy of the remnant liver and thus increase the safety of major hepatectomy^27^.

Different from previous studies^12,28^, the present study showed that the intraoperative bleeding and intraoperative blood transfusion had no significant difference between groups before and after matching, this may be due to AR are often performed under inflow occlusion of the liver by a Pringle maneuver^28^ in order to minimize bleeding during the transection phase. In addition, operation time were slightly decreased in the NAR group, but there were no significant difference between groups. Interestingly, in our current analysis, we found NAR group had more patients with tumour lesions located in the right liver than AR group, generally, the time spent at resection of the right liver tumour is longer than that of the left liver tomour, this might be explained by the fact that the right liver resection was more difficult than the left. That is, operation time in NAR group was less than in AR group. Furthermore, hepatic resection can be associated with a certain degree of peri-operative morbidity and mortality. In the current analysis, the length of hospital stay and the overall incidence of complications were largely comparable among patients who underwent AR versus NAR. Meanwhile, there were no significant differences of CDC I, CDC II, CDC III or CDC V between groups. However, notably, the risk of CDC IV in the AR group was significant higher than that in the NAR group. In addition, the incidence of postoperative hepatic failure, bile leakage and intra-abdominal bleeding as well as perioperative mortality did not differ between the two groups. Additionally, the occurrence of a postoperative complication has previously been reported to be an independent predictor of worse long-term outcomes^29^. However, we didn’t find the similar result, furthermore, a previous study^30^ found that the impact of major complications on survival was primarily in the immediate post-operative period rather than in the long term. Thus, these data suggest that the overall perioperative outcomes can be superior for NAR.

Regarding the clinicopathological factors affecting OS and DFS in patients with macroscopically solitary ICC lesions, univariate analysis showed that positivity of HBsAg was associated with worse DFS and OS. Thus, positivity for HBsAg was a risk factor for DFS and OS. However, in disagreement with a previous study^31^, this study showed antiviral therapy for HBV-related ICC patients didn’t improve survival outcomes. This maybe due to the comparable liver function or stable low level of virus reproduction. Consistent with previous studies^31,32^, vascular invasion was associated with worse OS, However, in the current study, the two groups had no significant difference of vascular invasion, thus, whether the AR is benefit for ICC patients with vascular invasion needs further study. Meanwhile, the results of our Cox regression analysis showed lymph node metastasis and adjuvant therapy were significant prognostic factors for OS and DFS, respectively. Similar to previous study^33,34^, lymph node metastasis was a risk factor for survival, however, there is no clear consensus on whether lymph nodes should be dissected^35,36^, and this aspect requires further research. Whilst the present study found the adjuvant therapy may improve the prognosis. Furthermore, previous study^37^ suggested that the neoadjuvant/adjuvant therapies may improve the prognosis of ICC patients especially for those with positive surgical margin. A meta-analysis^38^ demonstrated that adjuvant therapy benefited biliary cancer patients with node-positive or R1 disease.

In this study, the survival curves showed the NAR group had slightly improved DFS and OS than the AR group before and after matching, yet with no significant statistically differences. Similarly, a Japanese retrospective study^39^ had concluded that in HCC patients with impaired liver functions, limited liver resection without tumor exposure may provide longer tumor-free and overall survival. That is, the NAR had better survival outcomes than the AR especially for the patients with liver cirrhosis. In fact, several previous studies^23,40^ have noted that preservation of liver parenchyma should take priority, and minor hepatectomy can provide equal OS and DFS compared with major hepatectomy for small HCC.

In addition, some previous studies^41,42^ found that HCC and ICC tended more often to have associated micro-metastasis and disseminated via portal venous branches. Thus, local recurrence (LR) may be due to residual intrahepatic metastasis spreading through the portal venous system, which cannot be detected before and during surgery. AR based on the Couinaud system requires a complete removal of at least one Couinaud segment containing the tumour. This can completely eliminate the entire unilobar portal venous drainage of the involved lobe of the liver to decrease the LR. Pawlik T. M, *et.al*.^30^ found that patients undergoing major resection had similar outcomes to patients undergoing minor resection, as long as the surgical margin was wider than 5 mm. In contrast, when the surgical margin was narrow (1–4 mm), major resection was associated with a decrease in tumor recurrence compared with minor resection. Therefore, that is, both surgical margin and resection volume can impact the survival outcomes.

The current study found the NAR group had more patients with positive surgical margin, however, survival analysis showed that the surgical margin had no significant effect on DFS or OS before or after matching. Generally, the distance between surgical margin and lesion is greater in AR than in NAR; thus, theoretically, the NAR group had a higher potential of positive surgical margin than the AR group. Furthermore, previous studies^43,44^ reported that the residual tumor on the resection margin may grow and spread in a much more aggressive pattern, which may result in the early recurrence and poor OS. A systematic review and meta-analysis^45^ found that Patients with negative surgical margin had significantly favorable OS and progression-free survival after surgical resection for ICC. Similarly, Spolverato G, *et al*.^46^ had conclued, for patients undergoing resection of ICC, a positive margin was associated with an inferior long-term outcome. In addition, there was an incremental worsening DFS and OS as margin width decreased from 1 cm. Thus, one possible explanation for the results of the current study is that a higher application rate of adjuvant treatment in the NAR group. And as previous study^37^ mentioned, the patients with positive margin may benefit more from adjuvant therapy. In other words, the adjuvant therapy may improve the suvrvival of ICC patients especially for those with NAR. However, more studies are needed to validate these findings.

This study has several limitations, the main limitation is its retrospective design. And unmeasured differences between the 2 groups could potentially lead to confounding effects. The results are also limited by the small sample size. Thus, randomized controlled trials are needed to further determine whether NAR is superior to AR.

These findings suggest that ICC patients may receive more benefits from NAR than that from AR, especially for patients with liver cirrhosis due to the lower risk of CDC-IV such as liver failure. Thus, we conclude NAR was not inferior to AR in improving the survival outcomes for patients with primary solitary ICC lesions without direct invasion to contiguous organs or extrahepatic metastasis. Furthermore, patients may benefit from NAR.

## Methods

### Data Sources and Study Population

Data for all consecutive patients who underwent liver resection for primary solitary ICC lesions at West China Hospital of SiChuan University, China, from January 2007 to July 2017 were retrieved from a prospective database for this study. The Institutional Review Board of West China Hospital of SiChuan University approved this study, confirm that the study was performed in accordance with relevant guidelines/regulations and informed consent was obtained from all patients and/or their legal guardian/s. A total of 150 patients underwent initial, curative hepatectomy for histologically confirmed primary solitary ICC lesions without direct invasion to contiguous organs or extrahepatic metastasis. The resection margin was ascertained based on final pathology. Six patients died of some non-tumour-related causes during follow-up – 1 patient each died of intracerebral bleeding, severe pneumonia, gastrointestinal bleeding, acute cardiac infarction, liver failure, and car accident.

Data on standard demographic, perioperative clinicopathological, and tumour-related characteristics were collected. Factors compared in the AR and NAR groups included patient age and sex, positivity for HBsAg, intrahepatic biliary stones, Child-Pugh classification, carcinoembryonic antigen (CEA) level, carbohydrate antigen 19-9 (CA19-9) level, blood loss, blood transfusions, and complications. Tumour characteristics were based on final pathology reports, including liver cirrhosis, tumour size, tumor location, vascular/perineural/biliary/adjacent organ invasion, lymph node metastasis, and histological grade. Tumor size was defined as the largest diameter (in centimeters) for the tumor within the resected specimen. In addition, information on surgery, adjuvant therapy, such as adjuvant chemotherapy, radiofrequency ablation, transcatheter arterial chemoembolization, and re-operation was also collected for each patient. Margin status were determined from the final post-operative pathological report.

### Follow-up

After discharge, the patients were followed-up every 3 months for up to 2 years after the initial operation and every 6 months thereafter. Recurrence was defined as the appearance of a new lesion that exhibited radiologic features compatible with ICC. DFS was defined as the interval between the date of operation and the date of diagnosis of first recurrence or last follow-up. OS was measured from the date of operation to the date of death or last follow-up. The date of last follow-up and survival status were collected for all patients.

### Statistical analyses

Continuous data are expressed as medians and ranges. Qualitative variables are expressed as frequencies (percentages). The Student t test or Mann-Whitney U test was used for intergroup comparisons of quantitative variables as appropriate, whereas the χ2 test or Fisher exact test was used to compare categorical data. Two-sided P-values of <0.05 were considered statistically significant. A survival analysis was conducted using the Kaplan-Meier product-limit method, and the significance of differences between survival curves was determined using the log-rank test. Multivariate comparisons of survival distributions were carried out using Cox proportional hazard models. To eliminate selection bias, patients were matched 1:1 by propensity score^47,48^ based on their clinicopathological characteristics. All statistical analyses were performed using SPSS 23.0 (SPSS Inc., Chicago, IL).

## Electronic supplementary material

The distribution of tumor location in the AR and NAR groups

## Data Availability

The datasets generated during and/or analysed during the current study are available from the corresponding author on reasonable request.
