# Supplementary material for: Nonanatomic resection is not inferior to anatomic resection for primary intrahepatic cholangiocarcinoma: A propensity score analysis
Source: Sci Rep. 2018 Dec 12;8:17799. doi: 10.1038/s41598-018-35911-5 (PMC6290773; doi:10.1038/s41598-018-35911-5)
Supplement: Supplementary file 1 — The distribution of tumor location in the AR and NAR groups [file 41598_2018_35911_MOESM1_ESM.pdf]

Nonanatomic resection is not inferior to anatomic resection for primary intrahepatic cholangiocarcinoma: A propensity score analysis

Li Bo[1], Song Jiulin[1], Aierken Yiliyaer[1], Chen Yang[1], Zheng Jinli[1], Yang Jiayin[1*]

[1]Liver Transplantation Center, Department of Liver Surgery, West China Hospital of Sichuan University, Chengdu 610041, Sichuan Province, China.

[*]corresponding.author@email.yang_jy123@sina.com

Table S1

The distribution of tumor location in the AR and NAR groups

tumor location	AR (n=85)	NAR (n=65)	*P* value
			＜0.001*
Left liver	46 (54.1%)	8 (12.3%)	
Right liver	31.3 (36.5%)	48 (73.8%)	
Middle liver	8 (9.4%)	9 (13.8%)	

* Indicates statistically significant.

AR, anatomic resection; NAR, nonanatomic resection.
